# The murine lens: A model to investigate in vivo epithelial–mesenchymal transition

**DOI:** 10.1002/dvdy.24518

**Published:** 2017-06-01

**Authors:** Kumi Shirai, Sai‐ichi Tanaka, Frank J. Lovicu, Shizuya Saika

**Affiliations:** ^1^ Department of Ophthalmology Wakayama Medical University Wakayama Japan; ^2^ Save Sight Institute and Discipline of Anatomy and Histology, Bosch Institute, School of Medical Sciences The University of Sydney NSW Australia

**Keywords:** lens, myofibroblast, fibrosis, mouse

## Abstract

Epithelial–mesenchymal transition (EMT) produces myofibroblasts that contribute to the formation of fibrotic tissue with an impairment of tissue homeostasis and functionality. The crystalline lens of the eye is a unique transparent and isolated tissue. The lens vesicle becomes isolated from the surface ectoderm, its cells are all contained as they line the inner surface of the lens capsule. Clinically the formation of fibrotic tissue by the lens epithelial cells causes a type of cataract or opacification and contraction of the lens capsule postcataract surgery. Production of EMT in the intact animal lens by using specific gene transfer to the lens or experimental lens injury has been shown to be a powerful tool to investigate EMT processes. It is not easy to uncover whether the origin of the myofibroblast is epithelial cell‐derived or from other cell lineages in fibrotic tissues. However, myofibroblasts that appear in the crystalline lens pathology are totally derived from the lens epithelial cells for the reasons mentioned above. Here, we report on different animal models of lens EMT, using either transgenic approaches or injury to study the biological aspects of EMT. *Developmental Dynamics 247:340–345, 2018*. © 2017 The Authors Developmental Dynamics published by Wiley Periodicals, Inc. on behalf of American Association of Anatomists

## Key Findings

- Lens, Epithelial‐mesenchymal transition, mice.

## Tissue Fibrosis and Myofibroblasts

Tissue integrity is maintained by a complex interplay of cells and extracellular matrix (ECM). Upon injury or local inflammation, fibrotic or scarring tissue is formed to primarily heal the organ, followed by regeneration of its functionality (Gurtner et al., 2008). Various tissues/organs are susceptible to fibrotic diseases. Wound healing in a local tissue is performed by inflammation and activation of resident cells including fibroblasts both are well orchestrated to finalize the process with restoration of tissue homeostasis and functionality. However, disregulation (mainly over‐activation ) of local mesecnhymal cells (fibroblasts or myofibroblasts) could lead the formation of fibrotic tissue with an impairment of the organ function.

In general, myofibroblasts secrete ECM components to establish fibrotic tissue and exert a contractile force to the tissue (Gabbiani, 2003; Darby et al., 2014).

**Figure 1:**
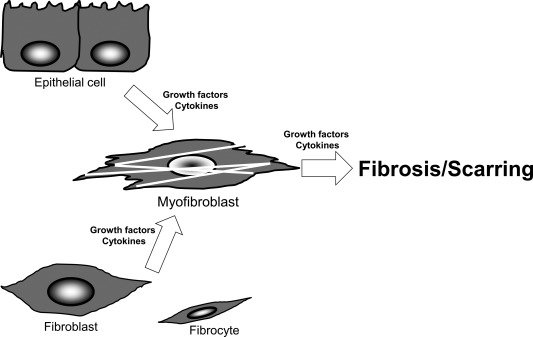
Origin of myofibroblasts in fibrotic lesions. Myofibroblasts can be derived from either a local fibroblast, an epithelial cell or sometimes from circulating bone‐marrow derived cells (fibrocytes), and play a central role in the formation of a fibrotic/scarring lesion. The myofibroblast has a critical role in the process of tissue fibrosis and extracellular matrix reconstruction. Accumulated matrix components and cytokines/chemokines expressed by infiltrated inflammatory cells further modulate myofibroblast generation and tissue fibrosis.

Myofibroblasts are involved in tissue fibrosis in lung (Willis et al., 2006), liver (Albanis and Friedman, 2001; Friedman, 2004), kidney (Sato et al., 2003; Zeisberg and Kalluri, 2004), skin (Darby et al., 2014), eye (retina [Bochaton‐Piallat et al., 2000; Saika et al, 2004a,2007a], lens [Saika et al., 2001; de Iongh et al., 2005; Shirai et al., 2006]) to name a few. Myofibroblasts that appear in the fibrotic lesion are considered to be a mixture of cells derived from either fibroblasts (Gabbiani, 2003; Hinz and Gabbiani, 2003; Micallef et al., 2012; Willis et al., 2006;), local epithelial cells (Kalluri and Neilson, 2003; Zeisberg and Kalluri, 2004), and bone‐marrow‐derived cells (Quan et al., 2006) (Fig. 1). However, in these pathological situations, the origin of myofibroblasts is not easily identified (Loeffler and Wolf, 2015; Sun et al., 2016).

Studies proposed the proportion of contribution of bone marrow‐derived cells (so‐called fibrocytes), local fibroblasts or epithelial cells, as the origin of myofibroblasts in fibrotic lesions in tissues. For example, in lung or kidney, alveolar epithelial cells or renal tubular epithelial cells are believed to supply myofibroblasts by means of EMT, and not bone marrow cells or local fibroblasts. However, different to the cell culture studies, it is quite difficult to uncover the precise contribution of EMT in fibrosis of these tissues (Kage and Borok, 2012; Noguchi et al., 2014; Loeffler and Wolf, 2015; Sun et al., 2016). Recent studies on tubulointerstitial fibrosis estimate that the origin of myofibroblasts are approximately 35% from fibroblasts that arise from the bone marrow, 10% and 5% by means of local EndoMT or EMT, respectively, and 50% from fibroblasts resulting from the proliferation of resident fibroblasts (Loeffler and Wolf, 2015). In contrast, the crystalline lens is a very unique tissue, with its cells totally isolated from other ocular tissues by a thickened basement membrane, the lens capsule. The lens epithelial cells line the inner surface of the anterior capsule and it is these same cells that undergo an EMT such that the resultant myofibroblasts, with accompanying ECM accumulation, leading to cataract are all of an epithelial origin.

Kalluri et al. define that EMT is a biologic process that allows a polarized epithelial cell to undergo multiple biochemical changes that enable it to assume a mesenchymal cell phenotype (Kalluri and Weinberg, 2009). They classified EMT into three types; Type I EMT (EMT during embryogenesis and organ development), Type II EMT (EMT associated with regeneration and fibrosis), and Type III EMT (EMT associated with cancer progression and metastasis). The process of production of myofibroblasts through EMT in tissue fibrosis that will be dealt in the current article is equivalent to Type II EMT (Kalluri and Neilson, 2003; Zeisberg and Kalluri, 2004; Willis et al., 2006).

The current article describes the usefulness of animal crystalline lens for the research in Type II EMT. The crystalline lens of the eye is a unique transparent and isolated tissue that is essential for vision. Surface head ectoderm invaginates with the eyecup of neuroectoderm origin to form the lens vesicle during embryonic development. Thus, as the lens vesicle becomes isolated from the surface ectoderm, its cells are all contained as they line the inner surface of the lens capsule, a thick special basement membrane. So the origin of all myofibroblasts produced by EMT in lens are lens epithelial cells.

## Cell Culture Studies on EMT

Investigators have conducted cell culture studies to reveal the roles of external conditions, i.e., growth factors/cytokines or extracellular matrix in the modulation of EMT and the generation of myofibroblasts from fibroblasts. One of the major growth factors/cytokines is the fibrogenic cytokine, transforming growth factor β (TGFβ), believed to be the most potent factor in promoting the process of EMT and fibroblast‐myofiboblast conversion, as well as fibrogenic gene expression (Xu et al., 2009; Biernacka et al., 2011). In rat lens epithelial explants, TGFβ was first shown to promote a myofibroblastic phenotype, indicative of an EMT (Liu et al., 1994; Hales et al., 1994).

TGFβ family members (TGFβ1 to β3) use the canonical Smad‐signaling pathway. Upon TGFβ binding to its receptor, a pair of transmembrane receptor serine‐threonine kinases are activated. Activated Smad2/3 proteins then partner with the common mediator, Smad4, and together they translocate to the nucleus where they modulate TGFβ‐dependent gene expression. Differences in the roles in gene expression regulation between Smad2 and Smad3 were investigated in cell culture experiments (Piek et al., 2001). Smad2/3 signals are inhibited by the action of Smad7, an inhibitory Smad that is up‐regulated by Smad2/3 signaling (Massague, 2012). Inhibitors of differentiation (Id2 and Id3), both up‐regulated by bone morphogenetic protein (BMP)‐7, also suppress Smad2/3 signaling (Saika et al., 2006). Like other growth factors, TGFβ can also activate non‐Smad cascades, i.e., mitogen‐activated protein kinases (MAP kinase), p38 MAP kinase, or the c‐Jun N‐terminal kinase (JNK) cascade (Massague, 2012; Mu et al., 2012). Investigations have revealed the detailed signal transduction system involved in TGFβ‐mediated EMT. Dependent on the different cell types used in the in vitro studies, the signaling cascade required for the process of EMT could differ (Gotzmann et al., 2004; Zavadil and Böttinger, 2005; Xu et al., 2009; Lim et al, 2011).

## Organ Culture Experiments

**Figure 2:**
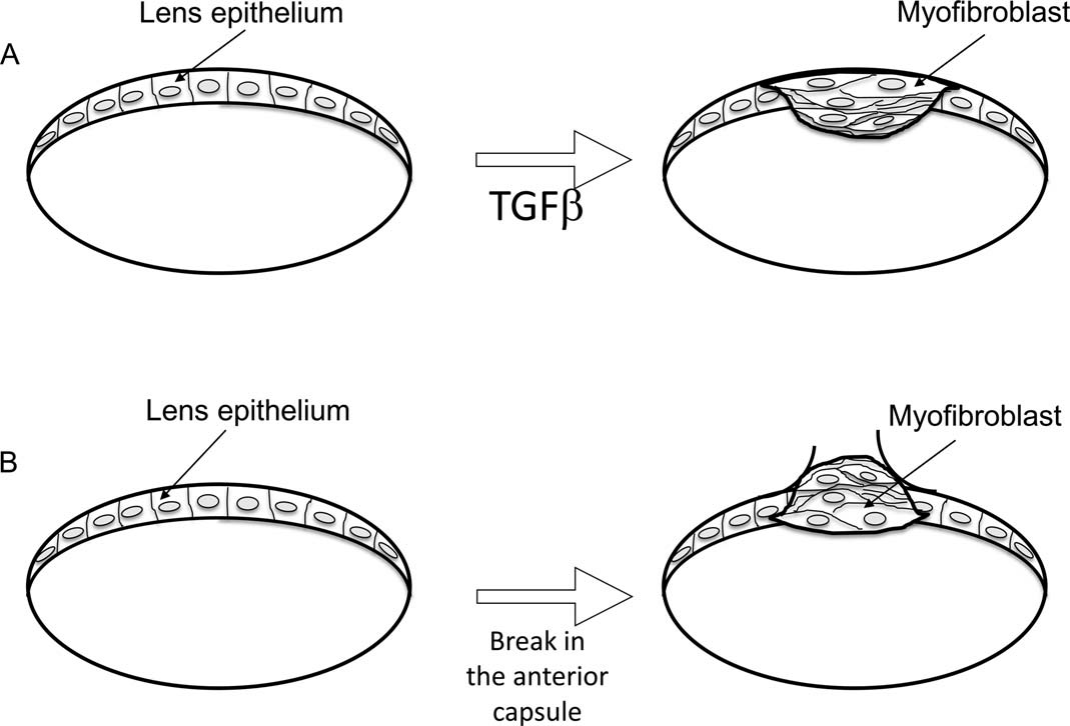
Development of myofibroblasts/fibrotic tissue in the lens. **A**: Lens epithelial cells of a rat or mouse can undergo EMT to produce myofibroblasts in organ‐culture or in situ with TGFβ. **B**: Alternatively, these myofbroblasts can form following an anterior capsular break injury in vivo. In both cases, there is no contamination by nonlenticular cell types.

It was reported that culturing the intact lens is a powerful tool to investigate lens epithelial cell behavior in situ. The procedure was originally developed using the rat lens (Hales et al., 1995), cultured whole in the presence of TGFβ to induce opacities that resulted from an EMT, with the appearance of myofibroblasts inside the lens capsule in place of lens epithelial cells (see Fig. 2A), indicating the effectiveness of exogenous factors on EMT on cells in their native setting. This work was nicely reviewed by de Iongh et al. (2005), with the procedure subsequently applied to investigating the intact lens of genetically modified mice displaying cataract. For example, adding TGFβ to the culture medium produces EMT‐type cataract in a rat lens in culture (Hales et al., 1995). We reported that this EMT‐cataract formation is attenuated by the loss of an ECM component (lumican) (Saika et al., 2003). Although EMT study in an organ‐cultured lens allowed us to study the cell behaviors in tissue, it prompted us to try to establish a strategy to study lens cell EMT in in vivo condition.

## In Vivo Study of EMT: Modification of Gene Expression in Murine Lens to Research EMT Processes

The role(s) of external factors in the modulation of EMT are examined in vivo for the purpose of investigating pathobiological mechanisms underlying disease. The major cellular component of scarring or fibrotic tissues is the myofibroblast, that exerts secretion of ECM components and contractile force (Gabbiani, 2003; Darby et al., 2014).

Delivery of TGFβ to lens cells can be extrinsic or intrinsic to the intact lens. Increased active TGFβ in the anterior ocular chamber, by direct injection of the ligand into the vitreous chamber, or adenoviral gene delivery, leads to EMT‐derived cataract, with fibrous tissue formation beneath the anterior capsule (Hales et al., 1999; Robertson et al., 2007). Compared with the strategy above, a more effective way to deliver this EMT inducer is to genetically modify the lens cells to overexpress active TGFβ in situ. Overexpressing a gene or the technique of gene knockout in the mouse lens is a refined way to uncover the role(s) of different lens EMT processes. The αA crystalline promoter was primarily used to drive gene expression in the lens of transgenic mice (Overbeek et al., 1985). Aberrant TGFβ overexpression in the lens using this promoter results in the induction of lens epithelial EMT to form a fibrous tissue containing myofibroblasts that model for human anterior subcapsular cataract (ASC) (Srinivasan et al., 1998; Lovicu et al., 2002).

In the Le‐Cre line, Cre‐recombinase is expressed in the developing murine lens, cornea, conjunctiva and skin of the eyelids from embryonic day 9 (Ashery‐Padan et al., 2000), while Cre‐recombinase expression in the MLR10‐Cre line is restricted to the differentiating lens epithelial and fiber cells (Zhao et al., 2004). Using either of these Cre lines, Lovicu et al. revealed that conditional deletion of the receptor tyrosine kinase inhibitors (Sprouty1 and Sprouty2) from the lens led to an elevation of ERK1/2 phosphorylation, together with the activation of aberrant TGFβ‐related signaling in lens epithelial cells, leading to an EMT and subsequent cataract formation (Shin et al., 2012), similar to that seen in transgenic mice overexpressing TGFβ in the lens (Lovicu et al., 2002). In turn, Sprouty overexpression in lens of transgenic mice was shown to suppress TGFβ‐induced EMT and cataract, highlighting the significant role of MAPK/ERK1/2‐signaling in EMT and cataract (Shin et al., 2012).

## Injury‐Induced EMT in Genetically Modified Mouse Lines

Another approach to investigate the roles of intrinsic components in the EMT process in the crystalline lens is to induce EMT‐based fibrotic cataract by external interventions on genetically modified mice. We showed that ocular surface alkali burn impacts not only the cornea but also the anterior chamber of the rat eye (Shirai et al., 2006), resulting in the lens epithelium undergoing an EMT leading to a fibrotic lesion inside the lens capsule (Shirai et al., 2006). Activation of TGFβ could be a main casdcade that lead to fibrotic lesion formation in the lens postocular surface alkali burn because the lesion was less severe in Smad3‐null mice. (Shirai et al., 2006).

**Figure 3:**
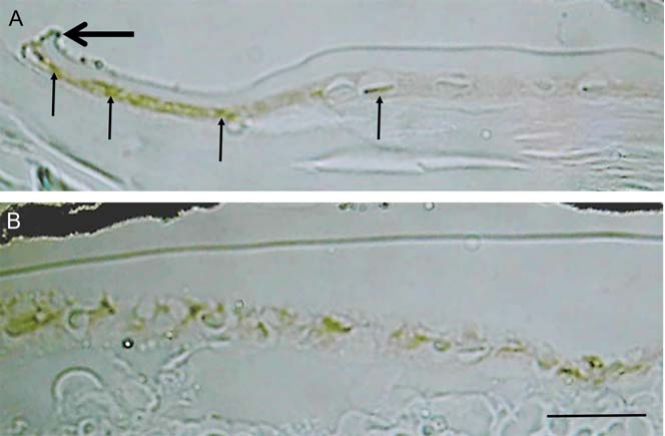
Smad4 signal is rapidly and transiently activated after breaking the anterior capsule in a mouse crystalline lens. **A,B**: At 12 hr postcapsular break (large arrow), Smad4 is accumulated in the lens cell nuclei (small arrows) beneath the anterior capsule (A), and by 24 hr nuclear translocation of Smad4 is readily detected in the mid‐peripheral area of these lens cells (B). Scale bar = 50 μm. (Reproduced from Shirai et al., 2014).

**Figure 4:**
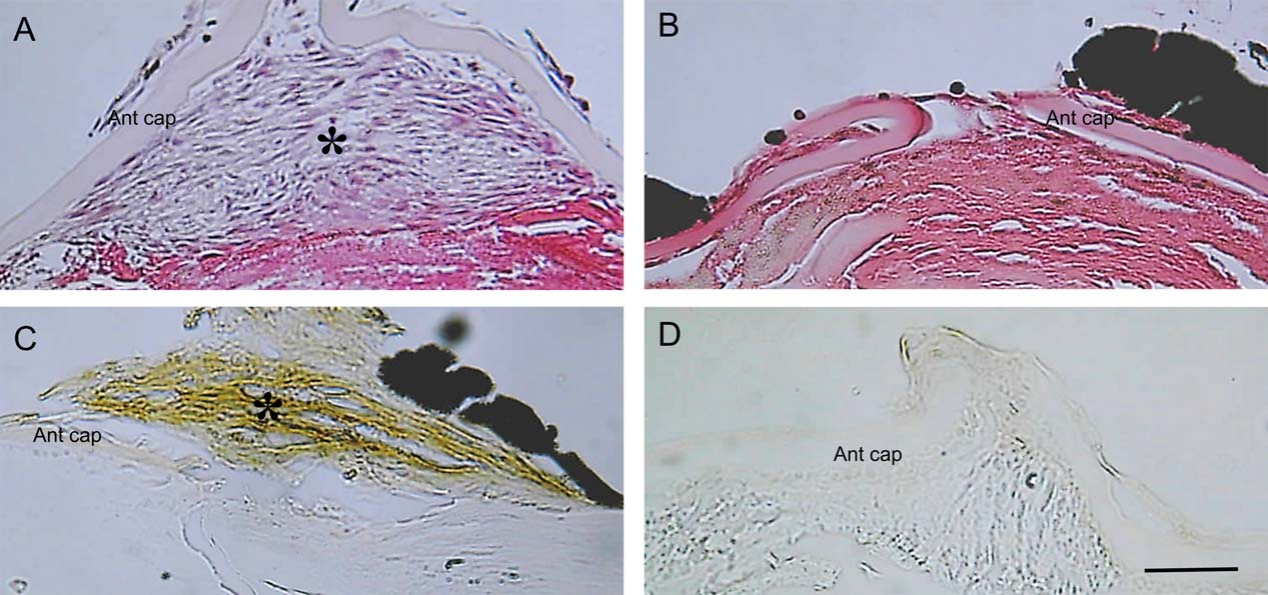
Appearance of myofibroblasts in an injured mouse lens is abolished by gene ablation of Smad3. **A**: At 4 weeks postpuncture injury of the anterior capsule of a wild‐type mouse lens, multilayered fibroblast‐like cells form (asterisk). **B**: Such a structure is not developed upon capsular injury in a Smad3‐deficient mouse lens. **C**: Immunohistochemistry reveals that cells in the wild‐type wounded lens are myofibroblasts reactive for α‐smooth muscle actin (asterisk). **D**: The monolayer of lens epithelial cells in the injured lens of the Smad3‐null mouse does not stain for α‐smooth muscle actin. Ant cap, anterior capsule. Scale bar = 50 μm. (Reproduced from Shirai et al., 2014).

EMT‐based fibrotic tissue is also formed following a puncture injury of the lens (Fig. 2B). Once the intact anterior lens capsule of mice is compromised, Smad is quickly and transiently activated in lens cells 12 hr postinjury (Saika et al., 2001) (Fig. 3). We showed that this Smad activation was abolished by intracameral injection of the anti‐TGFβ2 antibody at the time of capsular break (Saika et al., 2001). Although TGFβ/Smad signaling is considered the major signaling cascade involved in EMT of epithelial cell types, in vitro experiments have failed to clearly demonstrate the role of Samd2 or Smad3 in the process of EMT. To address this in vivo, the lens injury model was applied to Smad3‐null mice. The results of this showed that the loss of Smad3 blocks injury‐induced EMT in the mouse lens epithelium, in association with suppression of up‐regulation of downstream TGFβ‐signaling targets, such as Snail, as well as other EMT‐related components (Saika et al., 2004b) (Fig. 4).

EMT involves the accumulation of ECM components, leading to tissue fibrosis. In turn, the ECM molecules are also known to feedback and support the process of EMT by means of the modulation of growth factor signaling. Cell culture studies showed roles of ECM components in EMT. For example, fibronectin or collagen type I positively modulates the process of EMT in cultured cell types (Taliana et al., 2006; Shintani et al., 2008). As cultured cells may lose the native phenotype of their in vivo counterparts, and the extracellular micro‐environment in cell culture is quite different to the in vivo condition, it is, therefore, essential to investigate EMT in an in vivo setting, to better reproduce the pathobiology of disease.

Activated lens epithelial cells express various ECM components that contribute to the process of tissue repair and formation of fibrous tissue inside the crystalline lens. Such ECM components include collagen types I and III, fibronectin, tenascin C, or osteopontin. When the lens injury experiments were conducted in mouse lines that lack either tenascin C or osteopontin, in both these mouse lines, injury‐induced EMT in lens epithelium was markedly attenuated (Saika et al., 2007b; Tanaka et al., 2010). Cell culture studies showed that both tenascin C and osteopontin modulate TGFβ/Smad signaling, although the detailed mechanism of action might differ to each other; as the loss of osteopontin attenuates activation of Smad3 upon exposure to TGFβ in vitro (Saika et al., 2007b), while tenascin C might inhibit nuclear translocation of phsopho‐Smad (Carey et al., 2010). The lens injury model in mouse lines that lack either tenascin C or osteopontin exhibited attenuation of nuclear translocation of Smad3 with the delayed EMT (Saika et al., 2007b; Tanaka et al., 2010). Lacking specific molecular components in a genetically modified mouse line is a powerful tool to address their contribution to EMT of the lens epithelium.

## Gene Introduction to an Injured Mouse Lens

**Figure 5:**
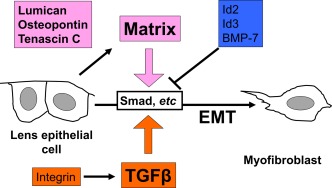
Modulating factors of EMT of lens epithelium. EMT of lens epithelium are progressed by TGFβ, extracellular matrix, and integrin, but are inhibited by bone morphogenic protein‐7 (BMP‐7) and signaling components induced by BMP‐7 (Id2 and Id3).

As discussed earlier, the injury‐induced appearance of myofibroblasts is totally dependent on the presence of lens epithelial cells. Therefore, gene transfer to this isolated tissue is suitable to evaluate the role of exogenous genes on the EMT of the lens epithelium. Gene transfer to an injured mouse lens using adenoviral vectors affected EMT of the lens epithelial cells. For example, we showed that adenoviral gene transfer of Smad7, the inhibitory Smad against Smads2/3, reproduced the effect seen in mice lacking Smad3, and blocks injury‐induced lens epithelial EMT (Saika et al., 2004c). Gene transfer of other anti‐Smad genes also exhibited an inhibitory effect on injury‐induced EMT in mouse lens epithelial cells in vivo. BMP‐7, a member of the TGFβ superfamily, counteracts TGFβ/Smads2/3 signals by inducing expression of Id2 and Id3 (Saika et al., 2006). BMP‐7 ligand or gene transfer, as well as gene transfer of Id2/3 inhibits EMT in vitro (Yang et al., 2016; Shu et al., 2017) and in vivo (Saika et al., 2006). Moreover, Integrins, cell surface receptors, play an important role in posterior capsular opacification (PCO). Fibrosis were inhibited in lens epithetial cells lacking αν integrins following surgical cell fiber cell removal (Mamuya et al., 2014). Smad3 phosphorylation was not detected in αν integrin null lenses (Mamuya et al., 2014). Integrins activate TGFβ and induce lens PCO and ASC (Walker and Menko, 2009) (Fig. 5).

In addition to its fundamental role in normal biological processes, clinically, inhibition of EMT by anti‐Smad strategies and anti‐integrin strategies is a potential therapeutic strategy to prevent the development of secondary cataracts that impair postoperative patients' vision, as residual lens epithelial cells undergo an EMT resulting in opacification of the lens capsule overlying the transparent prosthetic intraocular lens.

Overall, the murine lens is an ideal model to study EMT processes in situ and in vitro, owing primarily to its ease of manipulation and access, together with the fact that its thickened basement membrane isolates its cells, preventing any infiltration by extrinsic cells, but not the soluble molecules that readily traverse this barrier. By better understanding the defined EMT processes taking place in the lens, we will be able to extend these findings to other tissues, to hopefully one day develop specific treatments for ameliorating different systemic fibrotic diseases.

## Summary

EMT research by using an in vivo animal lens is a powerful tool to elucidate the pathophysiology and molecular biology underlying EMT by skipping a discussion on the origin of the cells, that also, in ophthalmology field, leads to the establishment of new strategies to overcome EMT type of cataract and capsular fibrosis postcataract surgery.
